# Effect of Health Education on Willingness to Undergo HIV Screening among Antenatal Attendees in a Teaching Hospital in North Central Nigeria

**DOI:** 10.1155/2014/456069

**Published:** 2014-06-30

**Authors:** O. O. Sekoni, S. A. Aderibigbe, T. M. Akande

**Affiliations:** ^1^Department of Preventive Medicine and Primary Care, College of Medicine, University of Ibadan, Ibadan, Nigeria; ^2^Department of Epidemiology and Community Health, University of Ilorin Teaching Hospital, P.M.B. 1459, Ilorin, Nigeria

## Abstract

*Background.* Testing for HIV during pregnancy provides a useful opportunity to institute treatment for HIV as required as well as protect the unborn baby. The aim of this study was to evaluate the effect of health education on the willingness of antenatal attendees to be screened for HIV. *Methods*. This was a quasiexperimental study involving the sequential enrolment of 122 pregnant women attending antenatal care who were at a gestational age of between 13 and 28 weeks for the study group and subsequent enrolment of the same one month after for the control. Two-stage analysis was done with the use of descriptive statistics and bivariate analysis. Level of significance was set at 5%. *Results*. Mean age of the study respondents was 27.6 ± 4.6 years while that of the control was 27.5 ± 4.8 years. Majority of the respondents were married in both study, 88 (72.7%), and control groups 84 (72.4%), 76.1% of the study group and 79.3% of the control group had at least secondary education, and 39.7% of the study group and 37.9% of the control group were primigravidae. Before intervention, 88.4% of the study group and 88.8% of the control group were willing to undergo voluntary HIV screening. There was an increase in this number after intervention (*P* < 0.05). Age, education, occupation, marital status, and parity were not significantly associated with a willingness to be screened for HIV before and after intervention among the study or control groups. *Conclusion*. Health education as a strategy to enhance voluntary counseling and testing uptake in antenatal settings is advocated.

## 1. Introduction

HIV/AIDS has been identified as one of the greatest scourges of mankind in the 21st century. AIDS is the fourth largest cause of death globally and the leading cause of death in Africa [1]. Affecting mainly the reproductive age group, it is a chronic illness which has a long incubation period, with many infected individuals including pregnant women not being aware of their HIV status [2, 3]. About 2.5 million people were newly infected with HIV/AIDS in 2011 alone, with 4.2 million adults and 800,000 children under 15 years [4]. At least 11 persons worldwide have been estimated to contact the disease every minute [5]. Over two-thirds of all the people now living with HIV in the world—nearly 21 million men, women, and children—live in Africa, and 83% of the world's AIDS deaths have been in this region [4]. Although infection with HIV/AIDS has been reported worldwide, sub-Saharan Africa bears the greatest brunt of the problem.

The epidemic in sub-Saharan Africa is driven by a host of factors including unprotected sex, poverty, lack of education and information, lack of access to modern medical facilities, exploitation of women, and regional armed conflict, as well as cultural, social, and economic issues. Women are more heavily affected in Africa than in other regions due to the fact that HIV has mostly spread through sex between men and women unlike other regions, where the virus initially spread most quickly among men by male to male sex or drug injecting. With its current estimated population of 130 million, Nigeria accounts for about 25 percent of the population in Africa. Nigeria is one of the most populous countries in the world to exceed 5 percent epidemiologically significant mark [6]. 3.4 million Nigerians were living with HIV/AIDS as of the year 2011 with only a small proportion of them knowing their HIV status [7, 8]. In many developing countries including Nigeria, data has shown that up to 60% of all new HIV infections are among the younger age group with females outnumbering the males in the ratio of two to one [9]. Knowing one's HIV status plays a very important role in bringing about behavioural change either to remain uninfected or to prevent the transmission of the infection to others [10, 11], and HIV screening serves as a very important strategy for the prevention of HIV/AIDS in Nigeria [12, 13]. Mother to child transmission (MTCT) of HIV is the commonest route of transmission of HIV/AIDS in infants, and this may occur antenatally, during labour, or postnatally through breast feeding [14]. Without treatment, HIV-positive mothers in developing countries face a 25–40% risk of giving birth to an HIV-positive child [9, 15]. HIV screening of pregnant women is a major step in the prevention and control of the HIV/AIDS epidemic.

The burden of HIV/AIDS can be reduced significantly using a 3-pronged intervention which includes voluntary counseling and confidential testing (VCCT) of antenatal patients, the provision of antiretroviral drugs (ARVs), and replacement feeding options for infants [16, 17]. The aim of this study took bearing from this evidence. Our study aimed to evaluate the effect of health education on the willingness to undergo HIV screening among pregnant women attending antenatal clinic in a university teaching hospital in north central Nigeria.

In Africa seroprevalence among pregnant women ranges from 5% to 35% with the highest rates among those in urban centres such as Abidjan, Blantyre, Kampala, and Lusaka [18]. While prepregnancy diagnosis of HIV is ideal, not all women have access to prepregnancy health care and, indeed, not all pregnancies are planned [2]. The benefits of early detection of HIV infection cannot be overemphasized in the prevention of mother to child transmission of HIV/AIDS. This is particularly so because infection of children through household contact is rare [19]. Pregnant women have been shown to express willingness to accept routine HIV screening once they have been adequately counseled about its benefits [20]. A previous study done among pregnant women attending antenatal care showed that 92.6% of those aware of HIV/AIDS were willing to be screened [21]. Though this is encouraging, it would indeed help to reduce the overall spread of HIV/AIDS if more individuals were willing to be screened. This is because, with a predominantly heterosexual mode of HIV transmission, it is necessary to identify infected pregnant women early through voluntary counseling and testing so that they can be given the option to take preventive drugs, which would, among other benefits, reduce mother to child transmission and help infected mothers stay healthy and productive for longer [22]. All behavioural changes necessary to end the HIV epidemic require individuals to take responsibility for their own health and for the health of others, including their loved ones, friends, and even strangers. HIV screening under the setting of a voluntary counseling and testing system is a key strategy for encouraging and empowering people to take these responsibilities.

This study will help assist policy makers on the issues to be considered in formulating HIV/AIDS policies especially for pregnant women. A good understudy of the willingness of pregnant women to undergo HIV screening will go a long way in assisting in the design and implementation of appropriate interventions.

## 2. Methodology

This was a quasiexperimental study conducted in three stages, namely, preintervention, intervention, and postintervention stages. The selection of the University of Ilorin Teaching Hospital for the study was due to the fact that facilities for both screening and confirmatory tests for HIV were available. The control group was equally selected from the University of Ilorin Teaching Hospital. For a comparison to be made to assess the effects of health education, the control group needs to be similar in all respects as the study group, and with only one tertiary health facility in the state, comparison with other primary and secondary health facilities would be inappropriate. Tertiary centres in neighbouring states have already implemented voluntary counseling and testing and/or routine HIV screening for all antenatal patients, which would serve as a bias in the study if such centres were used as control. Hence the use of the same health facility for both study and control was necessary.

Ilorin is made up of three local government areas, namely, Ilorin East, Ilorin West, and Ilorin South with their headquarters at Oke Oyi, Oja-Oba, and Fufu, respectively. Within the metropolis, there are many primary and secondary health institutions but only one tertiary health institution, namely, the University of Ilorin Teaching Hospital. There are many private health facilities scattered all over the city. This study was conducted at the maternity wing of the University of Ilorin Teaching Hospital. The hospital is located in Ilorin South local government area (LGA) of Kwara State. It serves Kwara State as a tertiary health facility for antenatal care, as well as its catchment areas of Kogi State, Niger State, and Osun State.

A minimum sample size of 122 was estimated using the formula by Kirkwood for the comparison of two proportions [23]. The formula utilized the proportion of pregnant women willing to be screened for HIV before health education intervention estimated at 62.9% [24] and those willing to be screened after health education intervention estimated at 79% [25]. Both of these proportions were obtained from published literature.

The hospital runs daily antenatal clinics from Monday to Friday, in which first time booking is done once a week while follow-up is carried out on the other four working days of the week. Appointments are given based on the gestational age of the pregnancy. While those whose gestational age is less than 28 weeks are seen monthly, those with gestational age between 28 and 36 weeks are seen fortnightly, and those with gestational age greater than 36 weeks are seen weekly. An average of 200–250 pregnant women book appointments at the antenatal clinic monthly. About 65% (130–162) of those that book appointments monthly fall within a gestational age of 13–28 weeks (obtained from hospital records). The total population of women at a gestational age of 13–28 weeks that came for booking within the time frame for recruitment of the study group (4 weeks) were recruited and sequentially selected until the desired sample size of 122 women was attained for the study group. The control group consisting of 122 women was similarly sequentially selected in the same way as the study group one month after the study group had been recruited. A pretested semistructured questionnaire was used for the study (Tables 2, 3, and 4). The pretest was carried out among 20 pregnant women attending antenatal clinic at Sobi Specialist Hospital, Ilorin (a secondary health facility), with a view for detecting deficiencies or ambiguities in the questionnaires and making appropriate corrections. It is located at the outskirts of the town along the major road that links Ilorin with Shao in Moro local government area.

EPI-INFO software version 6.0 was used for analysis. Two-stage analysis was done (analysis of the preintervention questionnaires and the postintervention questionnaires) with the use of descriptive statistics and bivariate analysis. A *P* value of less than 0.05 was considered as statistically significant.

## 3. Result

During the preintervention stage, 122 questionnaires each were administered to the study and control groups. Out of these, 121 (99.2%) were filled and returned for the study group while 116 (95.1%) were returned for the control group. They were analyzed after validation. After intervention, 122 questionnaires were administered to each group. Out of these, 117 (95.9%) were filled and returned for the study group while 114 (93.4%) were returned for the control group. They were analyzed after validation.

The respondents' ages ranged from 18 to 40 years. The mean age of the study respondents was 27.6 ± 4.6 years, and the modal age group was 20–29 years. The mean age of the control respondents was 27.5 ± 4.8 years, and the modal age group was 20–29 years (Table 1).

Majority of the respondents were married in both the study, 88 (72.7%), and the control groups, 84 (72.4%), with over half of the respondents being Muslims, while Christians constituted 43% and 30.2% of the study and control groups, respectively (Table 1).

Only 3 (2.4%) of the study group and 4 (3.5%) of the control group had no formal education at all, with about 76.1% of the study respondents and 79.3% of the control respondents having had at least secondary level of education (Table 1).

Majority of the respondents (86.8% of study group and 93.1% of control group) were Yoruba by ethnicity. Only 11 (9.1%) of the respondents from the study group and 6 (5.2%) respondents from the control group were from tribes other than the major tribes of Yoruba, Ibo, and Hausa in Nigeria and included tribes like the Nupe, Ebira, Igala, and the Tiv. Traders made up the largest group of the respondents in terms of occupation, with about 31.4% of them coming from the study group and 34.5% of them from the control group. Artisans and teachers made up 29.0% of the study group and 25.8% of the control group while students and housewives made up 29.8% of the study group and 29.3% of the control group (Table 1).

Less than half of the respondents (32.3% of the study group and 39.7% of the control group) had had at least three pregnancies, while 39.7% of the study group and 37.9% of the control group were primigravidae, and 28% of the study group and 22.4% of the control group were in their second pregnancy (Table 1).

Before intervention, 78.5% of the study respondents and 88.8% of the control respondents were willing to undergo voluntary HIV screening to know their HIV status. There was a significant increase in the number of study respondents who were willing to have HIV screening after intervention (*P* < 0.05). There was no significant difference among the control respondents (*P* > 0.05) (Table 5).

About 98.3% of the study respondents and 97.4% of the control respondents supported premarital HIV screening before intervention. There was no significant increase in the number of study respondents who supported premarital HIV screening after intervention or among the control respondents (*P* > 0.05). There was a significant increase after intervention in those who supported premarital screening as a form of protection for their partner as well as a way to prevent transmission to their children (*P* < 0.05) (Table 5).

Age, education, occupation, marital status, and parity were not found to be significantly associated with a willingness to be screened for HIV before and after intervention among the study group. There was also no significant association between these sociodemographic characteristics and a willingness to be screened among the control group (Table 6).

## 4. Discussion

Voluntary HIV screening is a strategy for encouraging and empowering people to take up positive health action and responsibilities, and this goes a long way to reduce the spread of the disease. About 78.5% of study respondents and 88.8% of control respondents expressed willingness to have voluntary HIV screening (Table 5). There was a significant increase in willingness to have voluntary HIV screening among the study respondents after intervention (*P* < 0.05). This finding compares favorably with a study done among pregnant women in Abeokuta, where up to 96% of the subjects were in support of routine HIV screening for pregnant women [20]. Similar results were found among pregnant women in Uganda, where almost all the respondents were willing to take an HIV test [26]. Preoperative patients have also been shown to express willingness to accept routine HIV screening [27]. Willingness for HIV testing may not always translate into actual use. Factors that have been associated with favourable response to HIV screening include confidentiality, presentation of VCT as routine rather than optional, and perceived high risk [28]. Testing rates among pregnant women have been found to be higher where an “opt-out” policy (in which women are informed that an HIV test is a standard part of antenatal care and that they may decline it) is in place than where an “opt-in” policy (in which women are required to specifically consent to an HIV test) is used [29].

Willingness to be screened was not significantly associated with the sociodemographic characteristics of age, education, marital status, occupation, or parity among the study respondents before or after intervention in this study. There was also no significant association between these sociodemographic characteristics and a willingness to be screened among the control group (Table 6). This suggests that, regardless of sociodemographic characteristics that exist, willingness to be screened may be majorly a function of information and education as to the benefits of such screening and not of independent human variables. Indeed, health education has been shown to bring about positive behavioural change with regard to HIV risk reduction [30].

As regards their response on whether intending couples should have premarital HIV screening (Table 5), over 95% of both the study and control respondents agreed that it should be done. On the other hand, this finding is at variance to a study on the perception of students in a tertiary institution in Ilorin, north central Nigeria, toward premarital screening for HIV, in which only 57.2% of the respondents expressed support for its enforcement [31]. The observed difference in the study among the pregnant women and the students is probably due to the setting of the studies and sociodemographic background of the respondents, which were quite different. While in this study most of the subjects were married and gainfully employed and all were attending antenatal clinic, the study carried out among students in the tertiary institution mentioned above involved subjects that were mostly single and unemployed and did not have any added motivation of the need to protect their unborn children.

Important advantages of premarital screening as expressed by the respondents in support of it revealed that 88.2% of the study group and 96.5% of the control group believed that it would serve to protect the other partner. There was a significant increase in the study respondents' view after intervention (*P* < 0.05). Similar views were expressed by students in a tertiary institution, with 73% of them identifying that it would prevent transmission to uninfected partner [31]. About 64.7% of the study group and 86.7% of the control group believed that it would serve to prevent transmission to their children by creating opportunity for intervention measures. There was a significant increase of this belief among the study respondents' after intervention (*P* < 0.05). These findings are in keeping with the principles guiding the use of screening as a public health tool in disease prevention [32] (Table 6).

## 5. Conclusions

This study looked at the willingness of pregnant women attending antenatal clinic in Ilorin to be screened for HIV. We acknowledge as a limitation the fact that our results may not be generalizable among all pregnant women since the study was conducted in just one health facility at one point in time. There is a need to replicate this study in other health facilities across the country as well as in other countries. In spite of this, our findings have implications for the design of intervention programs for the reduction of mother to child transmission of HIV. There was a significant increase in the willingness to be screened after intervention among the study group but not among the control group. It can be concluded that health education as a strategy to enhance voluntary counseling and testing uptake in antenatal settings is a viable option to be considered and is advocated. Further research into the benefits of premarital HIV screening is also recommended.

## Figures and Tables

**Table 1:** Sociodemographic characteristics of the respondents.

Variables	Study group (%) (*N* = 121)	Control group (%) (*N* = 116)
Age (in years)		
≤19	1 (0.8)	2 (1.7)
20–29	84 (69.4)	84 (72.4)
30–39	35 (29.0)	26 (22.4)
≥40	1 (0.8)	4 (3.5)
Marital status		
Single	24 (19.9)	19 (16.4)
Married	88 (72.7)	84 (72.4)
Cohabiting	9 (7.4)	13 (11.2)
Religion		
Christianity	52 (43.0)	35 (30.2)
Islam	69 (57.0)	81 (69.8)
Educational level		
None	3 (2.4)	4 (3.5)
Primary	26 (21.5)	20 (17.2)
Secondary	44 (36.4)	40 (34.5)
Postsecondary	48 (39.7)	52 (44.8)
Ethnic group		
Yoruba	105 (86.8)	108 (93.1)
Ibo	4 (3.3)	2 (1.7)
Hausa	1 (0.8)	0 (0.0)
Others	11 (9.1)	6 (5.2)
Occupation		
Artisan	25 (20.7)	18 (15.5)
Trading	38 (31.4)	40 (34.5)
Teaching	10 (8.3)	12 (10.3)
Student	23 (19.0)	26 (22.4)
Housewife	13 (10.8)	8 (6.9)
Others	12 (9.8)	12 (10.4)
Parity		
First time	48 (39.7)	44 (37.9)
Second time	34 (28.0)	26 (22.4)
Third time	14 (11.6)	25 (21.6)
More than three times	25 (20.7)	21 (18.1)

**Table 2:** Respondents' awareness of HIV/AIDS.

Ever heard of HIV/AIDS	Study group (%)		Control group (%)	
	Before	After	Before	After
	(*N* = 121)	(*N* = 117)	(*N* = 116)	(*N* = 114)
Yes	120 (99.2)	115 (98.3)	113 (97.4)	112 (98.2)
No	1 (0.8)	2 (1.7)	3 (2.6)	2 (1.8)

Total	121	117	116	114
*P* value	0.97622		0.98431	

**Table 3:** Knowledge of HIV-testing facility in the hospital.

Availability of HIV-testing services in this hospital	Study group (%)		Control group (%)	
	Before	After	Before	After
	(*N* = 121)	(*N* = 117)	(*N* = 116)	(*N* = 114)
Yes	79 (65.3)	112 (95.7)	41 (35.3)	40 (35.1)
No	15 (12.4)	0 (0.0)	2 (1.7)	2 (1.8)
Do not know	27 (22.3)	5 (4.3)	73 (62.9)	72 (63.2)

Total	121	117	116	114
*P* value	0.00000		0.99907	

**Table 4:** Distribution of respondents by perceived benefits of knowing HIV status.

Perceived benefits in knowing HIV status	Study group (%)		Control group (%)	
	Before	After	Before	After
	(*N* = 121)	(*N* = 117)	(*N* = 116)	(*N* = 114)
Yes	116 (95.9)	117 (100.0)	114 (98.3)	112 (98.2)
No	5 (4.1)	0 (0.0)	2 (1.7)	2 (1.7)

Total	121	117	116	114
*P* value	0.07669		0.62634	

**Table 5:** Willingness to have voluntary HIV screening.

Variable	Study group (%)		*P* value	Control group (%)		*P* value
	Before	After		Before	After	
	(*N* = 121)	(*N* = 117)		(*N* = 116)	(*N* = 114)	
Willingness to know HIV status						
Yes	95 (78.5)	111 (94.9)	0.0002	103 (88.8)	99 (86.8)	0.65
No	26 (21.5)	6 (5.1)		13 (11.2)	15 (13.2)	
Willingness to support premarital HIV screening						
Yes	119 (98.3)	115 (98.3)	0.64	113 (97.4)	111 (97.4)	0.69
No	2 (1.7)	2 (1.7)		3 (2.6)	3 (2.6)	
Reasons for support	(*N* = 119)	(*N* = 115)		(*N* = 113)	(*N* = 111)	
To protect partner						
Yes	105 (88.2)	112 (97.4)	0.01	109 (96.5)	104 (93.7)	0.34
No	14 (11.8)	3 (2.6)		4 (3.5)	7 (6.3)	
To prevent transmission to children						
Yes	77 (64.7)	108 (93.9)	0.001	98 (86.7)	96 (86.5)	0.96
No	42 (35.3)	7 (6.1)		15 (13.3)	15 (13.5)	

**Table 6:** Relationship between sociodemographic characteristics and willingness to be screened.

Sociodemographic characteristics	Study		Chi-square; degrees of freedom (df); *P* value	Control		Chi-square; df; *P* value
	Before	After		Before	After	
	*N* = 106 (%)	*N* = 104 (%)		*N* = 110 (%)	*N* = 102 (%)	
Age group						
≤19	1 (0.9)	1 (1.0)	*χ* ^2^ = 2.77; df = 3; *P* = 0.42874	0 (0.0)	1 (0.9)	*χ* ^2^ = 3.54 df = 3 *P* = 0.31569
20–29	75 (70.8)	77 (74.0)		74 (67.3)	76 (74.5)	
30–39	29 (27.4)	22 (21.2)		33 (30.0)	21 (20.7)	
≥40	1 (0.9)	4 (3.8)		3 (2.7)	4 (3.9)	
Education						
None	2 (1.9)	4 (3.9)	*χ* ^2^ = 1.78; df = 3; *P* = 0.61880	5 (4.6)	4 (3.9)	*χ* ^2^ = 1.90 df = 3 *P* = 0.59291
Primary	23 (21.7)	17 (16.3)		25 (22.7)	16 (15.7)	
Secondary	39 (36.8)	37 (35.6)		37 (33.6)	36 (35.3)	
Postsecondary	42 (39.6)	46 (44.2)		43 (39.1)	46 (45.1)	
Marital status						
Single	22 (20.8)	16 (15.4)	*χ* ^2^ = 2.13; df = 2; *P* = 0.34548	18 (16.4)	16 (15.7)	*χ* ^2^ = 1.19 df = 2 *P* = 0.55273
Married	76 (71.7)	75 (72.1)		83 (75.5)	73 (71.6)	
Cohabiting	8 (7.5)	13 (12.5)		9 (8.1)	13 (12.7)	
Occupation						
Artisan	22 (20.8)	14 (13.5)	*χ* ^2^ = 3.13; df = 5; *P* = 0.67994	24 (21.8)	14 (13.7)	*χ* ^2^ = 4.13 df = 5 *P* = 0.53111
Trading	34 (32.1)	35 (33.7)		37 (33.6)	34 (33.3)	
Teaching	9 (8.5)	10 (9.6)		9 (8.2)	10 (9.8)	
Student	20 (18.8)	26 (25.0)		18 (16.4)	25 (24.5)	
Housewife	11 (10.4)	8 (7.6)		11 (10.0)	8 (7.8)	
Others	10 (9.4)	11 (10.6)		11 (10.0)	11 (10.9)	
Parity						
First time	42 (39.6)	39 (38.0)	*χ* ^2^ = 3.67; df = 3; *P* = 0.29904	42 (38.1)	39 (38.2)	*χ* ^2^ = 3.40 df = 3 *P* = 0.33356
Second time	28 (26.4)	24 (23.1)		23 (20.9)	23 (22.5)	
Third time	14 (13.2)	24 (23.1)		17 (15.5)	23 (22.5)	
More than three	22 (20.8)	17 (15.8)		28 (25.5)	17 (16.8)	
